# To be, or not to be: the dilemma of immunotherapy for non-small cell lung cancer harboring various driver mutations

**DOI:** 10.1007/s00432-023-04919-4

**Published:** 2023-06-01

**Authors:** Ruoxue Cai, Hongyu Zhu, Ying Liu, Huanhuan Sha, Weiwei Peng, Rong Yin, Guoren Zhou, Ying Fang

**Affiliations:** 1 Department of Oncology, The Affiliated Cancer Hospital of Nanjing Medical University, Jiangsu Cancer Hospital, Jiangsu Institute of Cancer Research, Baiziting 42, Nanjing, 210009 People’s Republic of China; 2 Department of Thoracic Surgery, The Affiliated Cancer Hospital of Nanjing Medical University, Jiangsu Cancer Hospital, Jiangsu Institute of Cancer Research, Nanjing, 210009 People’s Republic of China; 3 Department of Thoracic Surgery, Jiangsu Key Laboratory of Molecular and Translational Cancer Research, Jiangsu Cancer Hospital & Nanjing Medical University Affiliated Cancer Hospital & Jiangsu Institute of Cancer Research, Nanjing, 210009 People’s Republic of China; 4 Department of Oncology, Jiangsu Cancer Hospital, Jiangsu Institute of Cancer Research, The Affiliated Cancer Hospital of Nanjing Medical University, Nanjing, 210009 People’s Republic of China

**Keywords:** Non-small cell lung cancer (NSCLC), Immunotherapy, Driver mutations, Common mutation, Rare mutation

## Abstract

**Introduction:**

Lung cancer is one of primary cancer type with high incidence and mortality, non-small cell lung cancer (NSCLC) is the most common type of lung cncer. For advanced lung cancer, traditional chemotherapy and targeted therapy become difficult to solve the dilemma of further progress. In recent years, with the clinical application of immunotherapy, the therapeutic strategy of lung cancer has changed dramatically. At present, immunotherapy has shown conspicuous efficacy in NSCLC patients with high expression of programmed death-ligand 1 (PD-L1) and high tumor mutational burden (TMB). The discovery of driver mutations brings delightful hope for targeted cancer therapy. However, it remains controversial whether immunotherapy can be used in NSCLC patients with these specific driver mutations.

**Method:**

This article summarized the latest research progresses of immunotherapy in advanced NSCLC. We paid close attention to the relevance of various driver mutations and immunotherapy in NSCLC patients, and summarized the predictive effects of several driver mutations and immunotherapy.

**Results:**

The mutations of KRAS, KRAS+TP53, EPHA (especially EPHA5), ZFHX3, ZFHX3+TP53, NOTCH, BRAF and LRP1B+FAT3 have potential to be used as biomarkers to predict the positive effectiveness of immunotherapy. ZFHX3, ZFHX3+TP53, STKII/LKB1+KEAP1+SMARCA4+PBRM1 mutations in LUAD patients get more positive effect in immunotherapy. While the mutations of EGFR, KEAP1, STKII/LKB1+KRAS, EML4-ALK, MET exon 14 skipping mutation, PBRM1, STKII/LKB1+KEAP1+SMARCA4+PBRM1, ERBB2, PIK3CA and RET often indicate poor benefit from immunotherapy.

**Conclusion:**

Many gene mutations have been shown to be associated with immunotherapy efficacy. Gene mutations should be combined with PD-L1, TMB, etc. to predict the effect of immunotherapy.

## Introduction

Lung cancer is one of the primary cancer types with high incidence and mortality, and non-small cell lung cancer (NSCLC) is the most common pathological type (Molina et al. 2008). With the emergence of adverse effects and the toxicity of chemotherapy drugs, the resistance of targeted therapy, immunotherapy has stepped onto the historical stage of NSCLC treatment. Immunotherapy improves the anti-tumor immunity of tumor microenvironment through stimulating or mobilizing immune system. The discovery of programmed cell death 1 (PD-1), programmed cell death 1 ligand 1 (PD-L1) and cytotoxic T lymphocyte-associated protein-4 (CTLA-4) has promoted the development of tumor immunotherapy. Immunotherapy includes immune checkpoint inhibitors (ICIs) (targeting PD-1, PD-L1, TIGIT, Tim-3, Lag-3, etc.) (Anderson et al. 2016; Chauvin and Zarour 2020), immune checkpoint agonists (targeting CD40, TLR7, TLR8) (Freed-Pastor et al. 2021; Byrne et al. 2021; Mullins et al. 2019), cancer vaccines (Enokida et al. 2021), CAR-T therapy and other strategies. These treatments can effectively enhance the anti-tumor immune effect and inhibit the tumor growth. However, the curative effect of immunotherapy varies from patient to patient. How to predict the curative effect of immunotherapy to guide the clinical application of immunotherapy has become a hot research topic.

Recent researches have indicated that the expression of PD-L1 (Herbst et al. 2014), tumor mutation load (TMB) (Rizvi et al. 2018), special gene mutation, tumor-infiltrating lymphocytes (TIL) (Lu et al. 2019), antigen presentation defects (Thompson et al. 2021), gene expression profiles (GEPs) (Wang et al. 2019) could be used as predictive biomarkers of immunotherapy efficacy. Among them, PD-L1 expression, TMB (≥ 10 mut/Mb), microsatellite instability (MSI-H) and mismatch repair deficient (MMR) have been approved by health regulatory agencies, which can serve as predictive biomarkers for immunotherapy of NSCLC patients (Hellmann et al. 2018a, b; Marabelle et al. 2020; Bodor et al. 2020; Rizvi et al. 2018). In addition, being as the composite biomarkers, PD-L1 and TMB have stronger predictive abilities than alone (Rizvi et al. 2018; Carbone et al. 2017). In recent years, studies have shown that the mutation of *TP53*, one of the tumor suppressor genes, was related to high PD-L1 expression and high TMB (Dong et al. 2017). Besides, patients with *TET1* mutation achieved longer progression-free survival (PFS) and overall survival (OS) after treated by immunotherapy than those without *TET1* mutation in various cancers (Wu et al. 2019). Gene mutations may have some connection with DNA damage and repair (DDR) pathway to improve tumor immunogenicity by accumulating false DNA damage reaction, which shows good curative effect in immunotherapy (Wang et al. 2018; Teo et al. 2018). These findings manifest that some gene mutations can be new promising biomarkers for immunotherapy in NSCLC patients. Therefore, this review summarized the researches of immunotherapy in NSCLC patients with driver mutations in recent years, focusing on their relationship.

Here, we defined mutation rate of more than 5% as common mutation and mutation rate of less than 5% as rare mutation.

## Common gene mutations and immunotherapy in NSCLC

### TP53

As the most frequently mutant gene with more than 30% incidence in NSCLC (Dearden et al. 2013), the relevance of *TP53* and immunotherapy has been extensively studied. Several researches revealed that *TP53* mutation was regularly in connection with increased PD-L1 expression and TMB (Dong et al. 2017; Herbst et al. 2016). It also facilitated CD8 + T-cell infiltrations (CD8 + TILs) which were the predominant effector population following treatment with anti-PD-1/PD-L1 immunotherapy (Topalian et al. 2015). All these discoveries uncovered that *TP53* mutation played a role in immune checkpoint inhibitor therapies.

Further, *TP53* mutation included missense mutation and nonsense mutation (Bouaoun et al. 2016). Compared with the wild type, *TP53* missense mutation showed significantly higher PD-L1 expression. And *TP53* missense mutation was associated with a superior response to ICIs than *TP53* nonsense mutation (Sun et al. 2020). But no clinical study has validated this outcome.

In addition, a meta-analysis which analyzed 19 studies that involved 6,084 patients with NSCLC found the *TP53* wild type was correlated with a remarkable higher overall survival (OS) than the *TP53* mutant type (Gu et al. 2016). Meanwhile, another analysis also identified that lung adenocarcinoma (LUAD) patients with *TP53* mutation carried a poorer prognosis in contrast with those *TP53* wild type (Wang and Sun 2017).

*So, TP53* mutation is related with worse prognosis, but the efficacy with immunotherapy is still unclear.

### EGFR

The incidence of *EGFR* mutation was about 27% in NSCLC (Dearden et al. 2013). According to the CheckMate 057, KEYNOTE 010 and POPLAR trials, PD-1 inhibitors prolong the OS compared with docetaxel in NSCLC patients. However, in subgroup analysis, there was no remarkable difference in *EGFR* mutant patients (Borghaei et al. 2015; Herbst et al. 2016; Fehrenbacher et al. 2016). Many studies have reverified above findings. Chee Khoon Lee has indicated that for NSCLC patients with EGFR mutation, those treated by immunotherapy did not possess any more significant survival benefits than those treated by chemotherapy in second and later lines of therapy (Lee et al. 2018). Namely, in *EGFR* mutant advanced NSCLC patients, there is no significant improvement in OS between immunotherapy and chemotherapy.

Another retrospective analysis has suggested that NSCLC patients with *EGFR* mutation were related with poor immunotherapy efficacy (Gainor et al. 2016). It observed a statistically inferior progression-free survival (PFS) and objective response rate (ORR) for *EGFR* mutant patients received anti-PD-1/PD-L1 immunotherapy compared with the *EGFR* wild type. Further exploring possible mechanisms, we learned that EGFR mutations were relevant with fewer CD8 + TILs (Akbay et al. 2013).

All these studies confirmed that *EGFR* mutation could be regarded as a negative biomarker to immune checkpoint inhibitors.

### KEAP1

*KEAP1* is a mutated gene with the third frequency in LUAD. Study has found the frequency of *KEAP1* mutation is more than 17% in LUAD (Cancer Genome Atlas Research Network 2014).

A study evaluated the information from TCGA data has shown that *KEAP1* mutation has negative prognostic effect on immunotherapy (Cheng et al. 2021). But it has been found that *KEAP1* mutation showed close association with lower TILs and cytotoxic T lymphocyte. It indicated that *KEAP1* mutation may be associated with lower tumor immunity (Cheng et al. 2021).

Furthermore, other researches have also confirmed that patients with *KEAP1* and *STK11* mutation have poor therapeutic effect with pembrolizumab (Aggarwal et al. 2020).

Based on Impower150 study, Howard Jack West et al. have found compared with the wild type, patients with *KEAP1* mutation treated by atezolizumab and/or bevacizumab with carboplatin/paclitaxel were associated with inferior OS and PFS (West et al. 2022).

### KRAS

The incidence of *KRAS* mutation is about 17% in NSCLC (Dearden et al. 2013). ICIs can reduce the death risk of NSCLC patients with KRAS mutation compared with chemotherapy (Lee et al. 2018). Studies found that immunotherapy had a greater therapeutic benefit for *KRAS* mutation than *KRAS* wild type in NSCLC patients (Lee et al. 2018; Mazieres et al. 2019). The research which retrospectively analyzed 88 advanced NSCLC patients receiving immunotherapy disclosed that patients with *KRAS* mutation in immunotherapy had longer PFS and OS than the wild type (Dong et al. 2017). In addition, according to a prospective analysis of Song et al. patients who have higher rates of *KRAS* mutations treated by immunotherapy obtained durable benefit (Song et al. 2020).

The underlying mechanism why patients with *KRAS* mutation might benefit from anti-PD-1/PD-L1 immunotherapy remains unclear. Some researches pointed out that it might because *KRAS*-mutant tumors had more tumor-infiltrating lymphocytes in the microenvironment and were almost always active. On top of this, *KRAS*-mutant NSCLC expressed more PD-L1, and as mentioned above, the high expression of PD-L1 was confirmed to be related to better therapeutic effect (Mazieres et al. 2019; Rizvi et al. 2018).

Generally, tumor suppressor genes, such as *TP53, KEAP1, STK11/LKB1, ATM* and *CDKN2A*, are the most frequently co-mutated genes with *KRAS *(Aredo et al. 2019; Lee et al. 2018; Skoulidis et al. 2015). Studies suggested that the PD-L1 expression in *KRAS* + *TP53* co-mutation are much higher than the single mutation of *KRAS *(Skoulidis et al. 2018; Dong et al. 2017). A recent study which contained 165 patients with *KRAS*-mutant NSCLC undergoing anti-PD-1/PD-L1 immunotherapy demonstrated that the co-mutation of *TP53* might be associated with higher response (Lee et al. 2018). It can be inferred that NSCLC patients with co-mutation of *KRAS* and *TP53* are more sensitive to immunotherapy.

Therefore, it is speculated that *KRAS* mutation and *KRAS* + *TP53* co-mutation will become the predictive biomarkers for immunotherapy in NSCLC.

### STK11/LKB1

*STK11/LKB1* mutations are prevalent in NSCLC with 9% incidence (Dearden et al. 2013). They are related to lower a PD-L1 expression and an intermediate or high TMB. According to research findings, The *STK11/LKB1*-mutant tumors revealed significantly lower ORR/PFS/OS to anti-PD-1/PD-L1 immunotherapy (Skoulidis et al. 2018). And another research also uncovered that patients who harbored *STK11* mutation treating with immunotherapy showed progress disease and the PFS was only 4.2 weeks (Kauffmann-Guerrero et al. 2020). But another research indicated that there was no direct link to poor ICIs outcomes and *STK11/LKB1* mutation (Di Federico et al. 2021).

As mentioned above, *STK11/LKB1* are particularly prevalent among KRAS-mutant tumors. *STK11/LKB1* loss directly promotes the formation of non-T-cell-inflamed tumor immune microenvironment in immune competent murine models of *KRAS*-mutant LUAD (Skoulidis et al. 2018). Numerous researches found the co-mutation of *KRAS* and *STK11/LKB1* in NSCLC patients receiving immunotherapy has been confirmed to be related to poor therapeutic effect (Di Federico et al. 2021; Skoulidis et al. 2018). Indeed, co-existence of both mutations is associated with more metastatic sites at diagnosis and a higher risk of brain metastases (Calles et al. 2015).

In conclusion, the co-mutation of *STK11/LKB1* and *KRAS* can be considered as a negative predictive marker for immunotherapy in NSCLC.

### EPHA

Ephrin A receptor (*EPHA*) is an important member in receptor tyrosine kinase family, and it is a key regulator of intercellular signal transduction in normal development and diseases. *EPHA3-7* are all common mutant genes in NSCLC (about 5%–15%) (Jamal-Hanjani et al. 2017; Campbell et al. 2016; Hellmann et al. 2018a, b). At present, studies have indicated that *EPHA* mutation is a new predictor that significantly prolonged PFS in NSCLC patients with immunotherapy, which may independently predict the clinical benefits of immunotherapy in NSCLC without being affected by other gene mutations. Like *ZFHX* mutation, the superior clinical efficacy to immunotherapy with EPHA mutation is mostly manifested in LUAD patients (Bai et al. 2020).

*EPHA5*, as a member of the Eph receptor family, is a common mutation in LUAD (Chen et al. 2020). Chen et al. have found that compared with the wild type, the *EPHA5* mutant significantly changed tumor microenvironment, and the TMB level increased. It was speculated that immunotherapy was an effective treatment to the *EPHA5* mutant patients. And this study also indicated that the survival time of LUAD patients with *EPHA5* mutation who received immunotherapy was more prolonged (Chen et al. 2020). It implies that *EPHA5* mutation is a promising positive biomarker for immunotherapy in NSCLC, especially in LUAD patients. More importantly, this research pointed out that although patients with *EPHA5* mutation and high TMB showed a longer OS than those with low TMB, the OS time of *EPHA5* wild-type patients with high TMB was the same as that of patients with low TMB. Therefore, detecting *EPHA5* mutation may be useful to prevent over-treatment of patients who choose immunotherapy only based on high TMB.

### ZFHX3

*ZFHX3*, namely zinc finger homeobox 3, is an inhibitor of alpha-fetoprotein gene and one of the tumor suppressor genes in many cancers (Hu et al. 2019; Walker et al. 2015). According to the COSMIC database, the mutant rate of *ZFHX3* is about 7–8%. It may be related to brain metastasis in lung cancer (Song et al. 2021). Recently, studies have suggested that NSCLC patients with ZFHX3 mutation had a good prognosis after immunotherapy, and their PFS and OS were significantly longer than those of *ZFHX3* wild-type patients, especially the *ZFHX3* mutated LUAD patients (Zhang et al. 2021b, a). Further analysis suggests that it may be due to the positive correlation between *ZFHX3* mutation and the previously mentioned immunotherapy biomarkers, such as TILs, TMB, DDR pathway in NSCLC, etc. At the same time, the study revealed that activated CD4 + T cells, dendritic cells (DCs) and M1 macrophages were more abundant in *ZFHX3* mutated LUAD patients.

In addition, Zhang et al. have indicated that the *ZFHX3* mutation predicted higher survival rate in NSCLC patients treated with immunotherapy. And patients with the co-mutation of *TP53* and *ZFHX3* had longer OS than those with TP53 mutation after immunotherapy (Zhang et al. 2021a, b).

All the above results indicate that *ZFHX3* mutation can be regarded as a valuable predictive biomarker for immunotherapy in NSCLC, and it shows a positive therapeutic effect, especially for LUAD patients with *ZFHX3* and TP53 co-mutation.

### SMARCA4

*SMARCA4* alterations include two categories: class 1 are truncating mutations, fusions, and homozygous deletion and class 2 are missense mutations (Chakravarty et al. 2017). According to a large retrospective study gathering data from three institutions, the prevalence of *SMARCA4* mutation in NSCLC is about 6% (Cancer Genome Atlas Research Network 2014). Another study gathered the information of 532 patients from the immunotherapy-treated cohort has found that the ORR/PFS/OS of *SMARCA4* mutant patients had no significant extension with immunotherapy therapeutic efficacy (Alessi et al. 2021).

But previously another large study has indicated that *SMARCA4* mutant tumors tended more to have lower PD-L1 expression and higher TMB. It moved forward to illuminate those patients with *SMARCA4* mutant seemed to obtain benefit from immunotherapy, despite the negative PD-L1 expression (Schoenfeld et al. 2020).

In addition, Alessi et al. have found that *STK11* or *KEAP1* mutation often co-mutated with *SMARCA4* in NSCLC patients (Alessi et al. 2021). But the co-mutations of *STK11*, *KEAP1* and *SMARCA4* were associated with the negative immunotherapy effect (Di Federico et al. 2021; Marinelli et al. 2020).

Therefore, the relationship of immunotherapy and *SMARCA4* mutation remains unclear and further research is needed.

### EML4-ALK

The incidence of *EML4-ALK* mutation in NSCLC is about 5.3% (Dearden et al. 2013). The POPLAR and ATLANTIC trails have indicated that anti-PD-1/PD-L1 immunotherapy had lower therapeutic effect on NSCLC patients with *EML4-ALK* mutation than those with wild type (Fehrenbacher et al. 2016; Garassino et al. 2018). And a retrospective analysis also observed a significant shorter PFS and objective response rate (ORR) in *EML4-ALK* mutant patients with anti-PD-1/PD-L1 immunotherapy compared with those with wild type (Gainor et al. 2016). There were a large number of clinical researches proved that ICIs are ineffective in NSCLC patients with *EML4-ALK* mutation. That was likely because *EML4-ALK* mutation was not related with increased effector T cells which adjusted anti-tumor immune responses, despite it was associated with high expression of PD-L1 (Pyo et al. 2020).

However, there was a case report showed that a *EML4-ALK* mutant patient treated twice with ICIs obtained remarkable curative effect which may because of high TMB and abundant CD8 + T-cell infiltration (Song et al. 2019).

Therefore, although *EML4-ALK* mutation was often related with negative immunotherapeutic effect, some patients with that mutation received good survival benefits.

### PTEN

PTEN gene mutation in NSCLC is about 5.1% (Dearden et al. 2013). Little research has reported the association between PTEN mutation and immunotherapy in patients with NSCLC. Previously, a study has manifested that *PTEN* mutation was associated with immunotherapy resistance through enhancing the expression of immunosuppressive cytokines and inhibiting autophagy (Peng et al. 2016). Multiple clinical trials have confirmed the relevance of *PTEN* and immunotherapy resistance.

A case report observed that a patient who showed negative effect to Nivolumab was detected *PTEN* mutation by next-generation sequencing. It suggested that *PTEN* mutation in tumors was associated with immunotherapy resistance (Teng et al. 2022). Besides, another case also reported a NSCLC patient with *PTEN* mutation obtained poor immunotherapy efficacy (Ren et al. 2022). So, *PTEN* mutation may be hopefully considered as a new biomarker to predict negative therapeutic effect to immunotherapy in NSCLC.

A retrospective cohort study from the European Thoracic Oncology Platform (ETOP) Lungscape Project found that *PTEN* mutation was related with the expression of PD-L1 ≥ 1% cut-off (Kerr et al. 2019). However, another case report revealed a metastatic NSCLC patient with *PTEN* mutation expressed a poor response to the ICIs, although it exhibited high TMB and PD-L1 (Parikh et al. 2018).

In contrast to that, a prospective analysis reported by Peng Song has proved that patients who obtained durable benefit by immunotherapy in NSCLC had higher rates of *PTEN* mutation and *TP53* + *PTEN* co-mutation, suggesting that patients with these gene mutations may achieve positive effect from immunotherapy. But there was no specific statistical correlation between these genetic mutations and long‐term benefit outcomes (Song et al. 2020).

Above all, *PTEN* mutation is often associated with negative immunotherapeutic effect, but it still needs more large-scale studies to verify this conclusion.

## Rare gene mutations and immunotherapy in NSCLC

### NOTCH

*NOTCH* family consists of four members, including *NOTCH1/ NOTCH2/ NOTCH3/ NOTCH4 *(Mumm and Kopan 2000). According to the COSMIC database, the mutant rate of *NOTCH* is just about 5%.

A study has demonstrated that *NOTCH* mutation reduced immune cell infiltration, such as myeloid-derived suppressor cells, tumor-associated macrophages and Tregs. And it also decreased the expression of PD-1, CTLA-4, TIM-3 and LAG-3 (Mao et al. 2018).

Kai Zhang et al. have detected the association between *NOTCH* mutation and positive immunotherapeutic clinical effect. The overall immunotherapy response rate was 20.7% in NSCLC patients with *NOTCH* mutation. In addition, the median PFS and OS were 3.1 months and 16.0 months, respectively (Zhang et al. 2020). It also found patients with *NOTCH1, NOTCH2* or *NOTCH3* mutations exerted longer ORR and PFS than the patients with *NOTCH* wild type, but patients with *NOTCH4* mutation did not have this trend (Zhang et al. 2020). However, another research found *NOTCH4* mutant tumors were characterized by the abundant expressions of TMB and high CD8 T-cell infiltration, which indicated the *NOTCH4* mutation may also be associated with good immunotherapy benefit (Long et al. 2021).

### MET

*MET* gene alteration existed in 3–4% of NSCLC. One of the gene mutations was *MET* exon 14 skipping mutation (Frampton et al. 2015; Awad et al. 2016).

A study enrolled 63 NSCLC patients with *MET* exon 14 skipping mutation, the duration of immunotherapy ranged from 2 weeks to 9.6 months and ORR was only 17%, the effect of immunotherapy was poor compared with that of targeted therapy which ORR was 32% and median PFS was 7.3 months (Drilon et al. 2020). Also, The ImmunoTarget multicentric worldwide retrospective study showed that 36 NSCLC patients with *MET* mutation reflected 16% ORR and the median PFS and OS were 3.4 months and 18.4 months, respectively (Mazieres et al. 2019). A recent study has also found patients treated with immune checkpoint blockade with *MET* mutation had short median PFS (only 2.69 months) (Negrao et al. 2021).

Another retrospective study recruited 147 patients with *MET* exon 14 skipping mutation in lung cancer has showed that responses of immunotherapy were related to neither high PD-L1 expression nor high TMB (Sabari et al. 2018).

### PBRM1

*PBRM1* is a tumor suppressor gene which regulates the cell cycle, maintains the stability of the genome and improves centromere cohesion (Mota et al. 2019). It has been found that *PBRM1* mutation was particularly common in renal clear cell carcinoma, and it has been proved that *PBRM1* mutation was considered as a significant biomarker for immunotherapy in renal clear cell carcinoma (Braun et al. 2019).

The relevance of *PBRM1* mutation and immunotherapy in lung cancer is still unclear. Recently, a large retrospective study gathering data from three institutions found the prevalence of *PBRM1* mutation in NSCLC was about 3.04% (Zhou et al. 2020). It also pointed out that the mutation of *PBRM1* often indicated poor efficacy of immunotherapy in NSCLC patients (Zhou et al. 2020). According to this study, patients with *PBRM1* mutation tended to have higher TMB, but in both the high TMB and low TMB groups, patients with *PBRM1* mutation who received immunotherapy had lower OS than those with wild type. Therefore, *PBRM1* is more likely to be a promising biomarker to forecast poor survival benefit of receiving immunotherapy.

Another study combined the date from 240 advanced NSCLC patients to find the relevance between *PBRM1* mutation and the PFS after treating with anti-PD-L1 immunotherapy. It also indicated that *PBRM1* mutant patients in LUAD tended to express higher TMB but a less PFS (Yang et al. 2021).

Moreover, studies have shown that two or more co-mutations often occurred in *KEAP1, LKB1/STK11, PBRM1* and *SMARCA4*, which were related to the decline of immunotherapy effect (Marinelli et al. 2020; Di Federico et al. 2021). This study revealed that the co-mutation of the above four genes showed higher TMB in LUAD. But the survival time is significantly less than those patients without these co-mutations. Therefore, when two or more gene mutations of *KEAP1, STK11, PBRM1* and *SMARCA4* coexist in NSCLC patients, especially in LUAD patients, it is still necessary to use immunotherapy with caution.

### ERBB2

*ERBB2* mutation occurs in 2–4% of NSCLC patients, more frequently in LUAD and never-smokers (Ekman 2019). Most of patients with *ERBB2* mutation were with in-frame insertions in exon 20 (*ERBB2-ex20ins*) mutation, which were found about 1.7% incidence in NSCLC patients (Mazières et al. 2013). Nowadays, the therapeutic effect of immunotherapy in NSCLC patients with *ERBB2* mutation was still unclear. What we already learned is that *ERBB2* amplified tumors were associated with higher TMB (Dudnik et al. 2018a, b), but PD-L1 expression was low (Guisier et al. 2020). Some researchers have speculated the negative efficacy of ICIs in *ERBB2-ex20ins* mutant patients might be attributed to lower cytotoxic CD8 + T-cell infiltration and lower PD-L1 expression (Gainor et al. 2016).

The ImmunoTarget multicentric worldwide retrospective study showed a negative therapeutic effect of anti-PD-1/PD-L1 immunotherapy in *ERBB2* mutation subgroups (Mazieres et al. 2019). A study which performed genomic profiling of 78 NSCLC patients has indicated that patients with *ERBB2* mutation manifested lower PFS than those with wild type (Fang et al. 2019). Another large retrospective analysis indicated that patients with *ERBB2* mutation have showed poor response to immunotherapy (Guisier et al. 2020).

However, a case report showed a patient with *ERBB2-ex20ins* mutation significantly benefited from anti-PD-1 therapy plus chemotherapy treatment and showed more than half of tumor reduction (Tian et al. 2021).

### BRAF

The incidence of *BRAF* mutation is about 2.5% in NSCLC (Dearden et al. 2013). *BRAF V600E* is uniformly considered as the most common type. It consists in more than half of the patients in *BRAF* mutation (Ding et al. 2017). At present, the significantly potential efficacy of *BRAF* mutation in immunotherapy in melanoma has already been suggested (Welsh et al. 2016).

According to a retrospective analysis, *BRAF* mutation in NSCLC is connected with higher PD-L1 expression, lower TMB and lower MS-Stable status. And ICIs are effective in NSCLC patients with both *BRAF V600E* and *non-V600E* mutations (Dudnik et al. 2018a, b). A large retrospective study indicated that *BRAF* mutation was related to a better immunotherapy effect. It has been found that patients with *BRAF* mutation might be considered for immunotherapy after targeted therapy and first-line chemotherapy (Mazieres et al. 2019). Another large retrospective analysis also proved that patients with *BRAF* mutation in immunotherapy have showed effective responses (Guisier et al. 2020).

### PIK3CA

The prevalence of *SMARCA4* mutation in NSCLC is about 2%. Many researches showed patients with *PIK3CA* mutation had poor immunotherapy effect.

A retrospective study including 84 NSCLC patients who were treated with immunotherapy analyzed the correlation of molecular findings and immunotherapy response. It indicated that all 5 patients existing *PIK3CA* mutation expressed low PFS to immunotherapy and showed minimal or even no PD-L1 expression (Kauffmann-Guerrero et al. 2020).

Besides, *PIK3CA* mutant LUSC exhibited substantially low expression of PD-L1 and its surrounding immune cells reduced the expression of PD-1 receptor compared with wild-type tumors (Choi et al. 2017). Meanwhile, Kadara et al. found the expression of PD-L1 was also significantly decreased in *PIK3CA* mutant LUAD (Kadara et al. 2017). Since objective response to atezolizumab was found to be remarkably associated with high expression of PD-L1, this tended to show that *PIK3CA* mutation might be a biomarker for negative response to immunotherapy (Herbst et al. 2014).

### ROS1

*ROS1* rearrangement was initially identified from the cell of glioblastoma (Birchmeier et al. 1987), and was first identified in NSCLC in 2007(Rikova et al. 2007). The incidence of *ROS1* rearrangement was about 1%–2% in NSCLC patients (Gainor and Shaw 2013).

There were fewer studies investigate the relationship of this gene mutation and immunotherapy. In a retrospective study, only one NSCLC patient treated with ICIs harboring *ROS1* rearrangement, and the PFS and OS were both only 0.1 month (Dudnik et al. 2018a, b).

According to a recent study, patients treated with immune checkpoint blockade with *ROS1* rearrangement had short PFS, although they expressed high PD-L1 (up to 55%) (Negrao et al. 2021). This suggested that there were oncogene-specific factors apart from PD-L1 expression influenced clinical immunotherapeutic effect. Thus, PD-L1 might not be independent predictors to immunotherapy effect (Negrao et al. 2021).

Therefore, ROS1 rearrangement was correlated with negative immunotherapeutic effect. But large-scale researches were needed to verify that.

### RET

*RET* fusion was also identified less than 5% (at approximately 1%-2%) frequency in NSCLC. And it was more prevalent among LUAD never-smokers (Takeuchi et al. 2012; Kohno et al. 2012). Multiple studies have evaluated that NSCLC patients with *RET* fusion had negative effect in ICIs.

Jiyun Lee et al. have found the median PFS of NSCLC patients with *RET* fusion treated by immunotherapy was only 2.1 months, and the ORR was just 7.7%. On the contrary, the ORR among patients treated with pemetrexed-based regimens was 63.0%, and the median PFS was 9.0 months (Lee et al. 2020). Besides, this study also found patients with *RET* fusion were more likely to develop intracranial metastases.

Meanwhile, a large retrospective multicenter study indicated that patient with *RET* mutation was only one and had showed negative response in immunotherapy (Guisier et al. 2020). And Marcelo V Negrao et al. have also found patients with RET fusion treated with immune checkpoint blockade had short PFS (Negrao et al. 2021).

### FBXW7

The *FBXW7* gene, lies at chromosome 4q31q.3, is also one of the tumor suppressor genes. No study has counted the incidence of *FBXW7*.

The clinical significance of its mutation is obvious in various cancers, such as lung, hematopoietic, colon, esophageal, gastric, etc., and it is closely associated with the occurrence of cancer, tumor metastasis, poor prognosis and drug resistance of adjuvant therapy (Fan et al. 2022; Yeh et al. 2018). Little is known as regards the treatment effect of immunotherapy in NSCLC patients with *FBXW7* mutation. At present, another research has found if *FBXW7* mutation existed in malignant melanoma, it would become resistant to immunotherapy (Gstalder et al. 2020).

The latest clinical research shows that the expressions of TMB and CD8 + T cells and macrophages in NSCLC patients with *FBXW7* mutation are significantly higher than those of patients with wild type. Patients can get better clinical benefits from immunotherapy (Liu et al. 2022). However, further research is needed to prove or overturn this conclusion.

### LRP1B and FAT3 co-mutation

*LRP1B* is one of the tumor suppressor genes which encodes low-density lipoprotein (LDL) family receptor (Liu et al. 2001). *FAT3* is also one of tumor suppressor genes, which is a part of *FAT* family genes encoding large proteins with extracellular Cadherin repeats, EGF-like domains, and Laminin G-like domains (Katoh 2012). At present, it has been found that *FAT3* often co-mutates with *LRP1B*. And the expression of TMB and CD8A in co-mutation showed higher level than single mutation (Zhu et al. 2021).

On the basis of TCGA dataset, a study has found *LRP1B* evidently had much more than 5% mutation frequency (34.78%, 176/506) in LUAD patients. And the frequency of *FAT3* mutation was 21.34% (108 out of 506) in LUAD. Co-mutation of *FAT3* and *LRP1B* happened in 10.87% (55 out of 506) LUAD patients (Zhu et al. 2021). Most importantly, this study also indicated that patients with co-mutation of *FAT3* and *LRP1B* showed remarkably longer PFS with immunotherapy than patients with single mutation. Therefore, the co-mutation of *FAT3* and *LRP1B* genes can become another promising biomarker for NSCLC with immunotherapy. However, there is no research to prove whether LUAD patients with only *FAT3* or *LRP1B* mutation can benefit from immunotherapy yet.

## Conclusion and perspectives

In conclusion, as these clinical studies mentioned in this article, several gene mutations have shown potential as biomarkers for immunotherapy in NSCLC (Table 1). The mutations of *KRAS, KRAS* + *TP53, EPHA* (especially *EPHA5*), *ZFHX3, ZFHX3* + *TP53, NOTCH, BRAF* and *LRP1B* + *FAT3* have potential to be used as biomarkers to predict the positive effectiveness of immunotherapy. More importantly, *ZFHX3, ZFHX3* + *TP53, STKII/LKB1* + *KEAP1* + *SMARCA4* + *PBRM1* mutations in LUAD patients get more positive effect in immunotherapy. While the mutations of *EGFR, KEAP1*, *STKII/LKB1* + *KRAS, EML4-ALK, MET exon 14 skipping mutation, PBRM1, STKII/LKB1* + *KEAP1* + *SMARCA4* + *PBRM1, ERBB2*, *PIK3CA* and *RET* often indicate poor benefit from immunotherapy. It is well known that the guidelines have clearly stated that *EGFR* mutant patients with NSCLC generally did not use immunotherapy. However, the current researches have not made a clear judgment on the predictive significance of the following common or rare mutant genes. The predicting significancy of mutations like *TP53, STKII/LKB1, PTEN, SMARCA4, ROS1, FBXW7, LRP1B* and *FAT3* with immunotherapy is still controversial, further studies are needed to find out the relationship between these mutations and immunotherapy. In addition, the co-mutation of *TP53* + *ZFHX3* and *FAT3* + *LRP1B* showed better effect in immunotherapy than single mutation (Tables 2 and 3).

**Table 1:** The relationship of gene mutations, PD-L1 expression and TMB

Driver mutations	Incidence	PD-L1 expression	TMB
TP53	30%	High	High
EGFR	27%	–	–
KEAP1	17%	–	–
KRAS	17%	High	–
STK11 /LKB1	9%	Low	Intermediate or high
EPHA	5–15%	–	High
ZFHX3	7–8%	–	High
SMARCA4	6%	Low	High
EML4-ALK	5.3%	High	High
PTEN	5.1%	High	High
NOTCH	5%	–	High
MET	3–4%	High	High
PBRM1	3.04%	–	High
ERBB2	2–4%	Low	High
BRAF	2.5%	High	Low
PIK3CA	2%	Low	–
ROS1	1–2%	High	–
RET	1–2%	–	–
FBXW7	No count	–	High
LRP1B and FAT3 co-mutation	No count	–	High

1. High or low is compared to the wild type in the cited literature

2. “-” means not mentioned in the cited literature in this article

**Table 2:** The relationship of common gene mutations and immunotherapy

	Driver mutations	Medicines	Sample size	Representative reference
Positive	*KRAS*	Pembrolizumab	179	Dong et al. (2017)
	*KRAS* + *TP53*	Nivolumab/pembrolizumab/atezolizumab	174	Skoulidis et al. (2018)
	*EPHA*	Anti-PD-(L)1 monotherapy	239	Chen et al. (2020)
	*ZFHX3*	Anti-PD-(L)1 monotherapy or in combination with anti-CTLA-4	350	Zhang et al. (2021a, b)
	*ZFHX3* + *TP53*	Anti-PD-(L)1 monotherapy or in combination with anti-CTLA-4	329	Zhang et al. (2021a, b)
	*EGFR*	Nivolumab/pembrolizumab/atezolizumab	3025	Lee et al. (2018)
Negative	*KEAP1*	Atezolizumab and/or bevacizumab with carboplatin/paclitaxel	1694	West et al. (2022)
	*STKII/LKB1* + *KRAS*	Nivolumab/pembrolizumab/atezolizumab	174	Skoulidis et al. (2018)
	*EML4-ALK*	Atezolizumab	142	Fehrenbacher et al. (2016)

**Table 3:** The relationship of rare genes and immunotherapy

	Driver mutations	Medicines	Sample size	Reference
Positive	*NOTCH*	Anti-PD-(L)1 monotherapy	58	Zhang et al. (2020)
	*BRAF*	Nivolumab/ pembrolizumab/ atezolizumab	39	Dudnik et al. (2018a, b)
	*LRP1B* + *FAT3*	Immune checkpoint inhibitors	506	Zhu et al. (2021)
Negative	*MET exon 14* skipping mutation	Pembrolizumab/ nivolumab/ durvalumab/ atezolizumab/ ipilimumab + nivolumab	147	Sabari et al. (2018)
	*PBRM1*	Anti-PD-(L)1 monotherapy	240	Yang et al. (2021)
	*ERBB2*	Anti-PD-(L)1 monotherapy	78	Fang et al. (2019)
	*STKII* + *KEAP1* + *SMARCA4* + *PBRM1*	Anti-PD-(L)1 monotherapy	1269	Marinelli et al. (2020)
	*PIK3CA*	Nivolumab/atezolizumab/pembrolizumab	84	Kauffmann-Guerrero et al. (2020)
	*RET*	Nivolumab/pembrolizumab/atezolizumab/durvalumab/tremelimumab	59	Lee et al. (2020)

This paper may provide guidance for the appliance of immunotherapy in NSCLC patients. Except *EGFR* mutation which is fully studied in various researches, more large-scale clinical studies for positive mutations are needed to guide clinical treatments. Furthermore, gene mutations should be combined with PD-L1, TMB, TILs, etc. to predict the effect of immunotherapy. There should not only consider one factor.

## Data Availability

Not applicable.

## References

[CR1] Aggarwal C, Thompson JC, Chien AL, Quinn KJ, Hwang WT, Black TA, Yee SS, Christensen TE, LaRiviere MJ, Silva BA, Banks KC, Nagy RJ, Helman E, Berman AT, Ciunci CA, Singh AP, Wasser JS, Bauml JM, Langer CJ, Cohen RB, Carpenter EL (2020) Baseline plasma tumor mutation burden predicts response to pembrolizumab-based therapy in patients with metastatic non-small cell lung cancer. Clin Cancer Res 26:2354–2361 32102950 10.1158/1078-0432.CCR-19-3663 PMC7231655

[CR2] Akbay EA, Koyama S, Carretero J, Altabef A, Tchaicha JH, Christensen CL, Mikse OR, Cherniack AD, Beauchamp EM, Pugh TJ, Wilkerson MD, Fecci PE, Butaney M, Reibel JB, Soucheray M, Cohoon TJ, Janne PA, Meyerson M, Hayes DN, Shapiro GI, Shimamura T, Sholl LM, Rodig SJ, Freeman GJ, Hammerman PS, Dranoff G, Wong KK (2013) Activation of the PD-1 pathway contributes to immune escape in EGFR-driven lung tumors. Cancer Discov 3:1355–1363 24078774 10.1158/2159-8290.CD-13-0310 PMC3864135

[CR3] Alessi JV, Ricciuti B, Spurr LF, Gupta H, Li YY, Glass C, Nishino M, Cherniack AD, Lindsay J, Sharma B, Felt KD, Rodig SJ, Cheng ML, Sholl LM, Awad MM (2021) SMARCA4 and other SWItch/sucrose nonfermentable family genomic alterations in NSCLC: clinicopathologic characteristics and outcomes to immune checkpoint inhibition. J Thorac Oncol 16:1176–1187 33845210 10.1016/j.jtho.2021.03.024

[CR4] Anderson AC, Joller N, Kuchroo VK (2016) Lag-3, Tim-3, and TIGIT: co-inhibitory receptors with specialized functions in immune regulation. Immunity 44:989–1004 27192565 10.1016/j.immuni.2016.05.001 PMC4942846

[CR5] Aredo JV, Padda SK, Kunder CA, Han SS, Neal JW, Shrager JB, Wakelee HA (2019) Impact of KRAS mutation subtype and concurrent pathogenic mutations on non-small cell lung cancer outcomes. Lung Cancer 133:144–150 31200821 10.1016/j.lungcan.2019.05.015 PMC9348589

[CR6] Awad MM, Oxnard GR, Jackman DM, Savukoski DO, Hall D, Shivdasani P, Heng JC, Dahlberg SE, Jänne PA, Verma S, Christensen J, Hammerman PS, Sholl LM (2016) MET exon 14 mutations in non-small-cell lung cancer are associated with advanced age and stage-dependent MET genomic amplification and c-met overexpression. J Clin Oncol 34:721–730 26729443 10.1200/JCO.2015.63.4600

[CR7] Bai H, Duan J, Li C, Xie W, Fang W, Xu Y, Wang G, Wan R, Sun J, Xu J, Wang X, Fei K, Zhao Z, Cai S, Zhang L, Wang J, Wang Z (2020) EPHA mutation as a predictor of immunotherapeutic efficacy in lung adenocarcinoma. J Immunother Cancer 8:e001315 33303576 10.1136/jitc-2020-001315 PMC7733211

[CR8] Birchmeier C, Sharma S, Wigler M (1987) Expression and rearrangement of the ROS1 gene in human glioblastoma cells. Proc Natl Acad Sci USA 84:9270–9274 2827175 10.1073/pnas.84.24.9270 PMC299735

[CR9] Bodor JN, Boumber Y, Borghaei H (2020) Biomarkers for immune checkpoint inhibition in non-small cell lung cancer (NSCLC). Cancer 126:260–270 31691957 10.1002/cncr.32468 PMC7372560

[CR10] Borghaei H, Paz-Ares L, Horn L, Spigel DR, Steins M, Ready NE, Chow LQ, Vokes EE, Felip E, Holgado E, Barlesi F, Kohlhäufl M, Arrieta O, Burgio MA, Fayette J, Lena H, Poddubskaya E, Gerber DE, Gettinger SN, Rudin CM, Rizvi N, Crinò L, Blumenschein GR Jr, Antonia SJ, Dorange C, Harbison CT, Graf Finckenstein F, Brahmer JR (2015) Nivolumab versus docetaxel in advanced nonsquamous non-small-cell lung cancer. N Engl J Med 373:1627–1639 26412456 10.1056/NEJMoa1507643 PMC5705936

[CR11] Bouaoun L, Sonkin D, Ardin M, Hollstein M, Byrnes G, Zavadil J, Olivier M (2016) TP53 variations in human cancers: new lessons from the IARC TP53 database and genomics data. Hum Mutat 37:865–876 27328919 10.1002/humu.23035

[CR12] Braun DA, Ishii Y, Walsh AM, Van Allen EM, Wu CJ, Shukla SA, Choueiri TK (2019) Clinical validation of PBRM1 alterations as a marker of immune checkpoint inhibitor response in renal cell carcinoma. JAMA Oncol 5:1631–1633 31486842 10.1001/jamaoncol.2019.3158 PMC6735411

[CR13] Byrne KT, Betts CB, Mick R, Sivagnanam S, Bajor DL, Laheru DA, Chiorean EG, O’Hara MH, Liudahl SM, Newcomb C, Alanio C, Ferreira AP, Park BS, Ohtani T, Huffman AP, Väyrynen SA, Dias Costa A, Kaiser JC, Lacroix AM, Redlinger C, Stern M, Nowak JA, Wherry EJ, Cheever MA, Wolpin BM, Furth EE, Jaffee EM, Coussens LM, Vonderheide RH (2021) Neoadjuvant selicrelumab, an agonist CD40 antibody, induces changes in the tumor microenvironment in patients with resectable pancreatic cancer. Clin Cancer Res 27:4574–4586 34112709 10.1158/1078-0432.CCR-21-1047 PMC8667686

[CR14] Calles A, Sholl LM, Rodig SJ, Pelton AK, Hornick JL, Butaney M, Lydon C, Dahlberg SE, Oxnard GR, Jackman DM, Jänne PA (2015) Immunohistochemical loss of LKB1 is a biomarker for more aggressive biology in KRAS-mutant lung adenocarcinoma. Clin Cancer Res 21:2851–2860 25737507 10.1158/1078-0432.CCR-14-3112

[CR15] Campbell JD, Alexandrov A, Kim J, Wala J, Berger AH, Pedamallu CS, Shukla SA, Guo G, Brooks AN, Murray BA, Imielinski M, Hu X, Ling S, Akbani R, Rosenberg M, Cibulskis C, Ramachandran A, Collisson EA, Kwiatkowski DJ, Lawrence MS, Weinstein JN, Verhaak RG, Wu CJ, Hammerman PS, Cherniack AD, Getz G, Artyomov MN, Schreiber R, Govindan R, Meyerson M (2016) Distinct patterns of somatic genome alterations in lung adenocarcinomas and squamous cell carcinomas. Nat Genet 48:607–616 27158780 10.1038/ng.3564 PMC4884143

[CR16] Carbone DP, Reck M, Paz-Ares L, Creelan B, Horn L, Steins M, Felip E, van den Heuvel MM, Ciuleanu TE, Badin F, Ready N, Hiltermann TJN, Nair S, Juergens R, Peters S, Minenza E, Wrangle JM, Rodriguez-Abreu D, Borghaei H, Blumenschein GR Jr, Villaruz LC, Havel L, Krejci J, Corral Jaime J, Chang H, Geese WJ, Bhagavatheeswaran P, Chen AC, Socinski MA (2017) First-line nivolumab in stage IV or recurrent non-small-cell lung cancer. N Engl J Med 376:2415–2426 28636851 10.1056/NEJMoa1613493 PMC6487310

[CR17] Chakravarty D, Gao J, Phillips SM, Kundra R, Zhang H, Wang J, Rudolph JE, Yaeger R, Soumerai T, Nissan MH, Chang MT, Chandarlapaty S, Traina TA, Paik PK, Ho AL, Hantash FM, Grupe A, Baxi SS, Callahan MK, Snyder A, Chi P, Danila D, Gounder M, Harding JJ, Hellmann MD, Iyer G, Janjigian Y, Kaley T, Levine DA, Lowery M, Omuro A, Postow MA, Rathkopf D, Shoushtari AN, Shukla N, Voss M, Paraiso E, Zehir A, Berger MF, Taylor BS, Saltz LB, Riely GJ, Ladanyi M, Hyman DM, Baselga J, Sabbatini P, Solit DB, Schultz N (2017) OncoKB: a precision oncology knowledge base. JCO Precis Oncol. 10.1200/PO.17.00011 28890946 10.1200/PO.17.00011 PMC5586540

[CR18] Chauvin JM, Zarour HM (2020) TIGIT in cancer immunotherapy. J Immunother Cancer 8:e000957 32900861 10.1136/jitc-2020-000957 PMC7477968

[CR19] Chen Z, Chen J, Ren D, Zhang J, Yang Y, Zhang H, Mao B, Ma H (2020) EPHA5 mutations predict survival after immunotherapy in lung adenocarcinoma. Aging (albany NY) 13:598–618 33288738 10.18632/aging.202169 PMC7834994

[CR20] Cheng W, Xu B, Zhang H, Fang S (2021) Lung adenocarcinoma patients with KEAP1 mutation harboring low immune cell infiltration and low activity of immune environment. Thorac Cancer 12:2458–2467 34328274 10.1111/1759-7714.14089 PMC8447911

[CR21] Choi M, Kadara H, Zhang J, Parra ER, Rodriguez-Canales J, Gaffney SG, Zhao Z, Behrens C, Fujimoto J, Chow C, Kim K, Kalhor N, Moran C, Rimm D, Swisher S, Gibbons DL, Heymach J, Kaftan E, Townsend JP, Lynch TJ, Schlessinger J, Lee J, Lifton RP, Herbst RS, Wistuba II (2017) Mutation profiles in early-stage lung squamous cell carcinoma with clinical follow-up and correlation with markers of immune function. Ann Oncol 28:83–89 28177435 10.1093/annonc/mdw437 PMC6246501

[CR22] Cancer Genome Atlas Research Network (2014) Comprehensive molecular profiling of lung adenocarcinoma. Nature 511:543–550 10.1038/nature13385 PMC4231481 25079552

[CR23] Dearden S, Stevens J, Wu YL, Blowers D (2013) Mutation incidence and coincidence in non small-cell lung cancer: meta-analyses by ethnicity and histology (mutMap). Ann Oncol 24:2371–2376 23723294 10.1093/annonc/mdt205 PMC3755331

[CR24] Di Federico A, De Giglio A, Parisi C, Gelsomino F (2021) STK11/LKB1 and KEAP1 mutations in non-small cell lung cancer: prognostic rather than predictive? Eur J Cancer 157:108–113 34500370 10.1016/j.ejca.2021.08.011

[CR25] Ding X, Zhang Z, Jiang T, Li X, Zhao C, Su B, Zhou C (2017) Clinicopathologic characteristics and outcomes of Chinese patients with non-small-cell lung cancer and BRAF mutation. Cancer Med 6:555–562 28135039 10.1002/cam4.1014 PMC5345676

[CR26] Dong ZY, Zhong WZ, Zhang XC, Su J, Xie Z, Liu SY, Tu HY, Chen HJ, Sun YL, Zhou Q, Yang JJ, Yang XN, Lin JX, Yan HH, Zhai HR, Yan LX, Liao RQ, Wu SP, Wu YL (2017) Potential predictive value of TP53 and KRAS mutation status for response to PD-1 blockade immunotherapy in lung adenocarcinoma. Clin Cancer Res 23:3012–3024 28039262 10.1158/1078-0432.CCR-16-2554

[CR27] Drilon A, Clark JW, Weiss J, Ou SI, Camidge DR, Solomon BJ, Otterson GA, Villaruz LC, Riely GJ, Heist RS, Awad MM, Shapiro GI, Satouchi M, Hida T, Hayashi H, Murphy DA, Wang SC, Li S, Usari T, Wilner KD, Paik PK (2020) Antitumor activity of crizotinib in lung cancers harboring a MET exon 14 alteration. Nat Med 26:47–51 31932802 10.1038/s41591-019-0716-8 PMC8500676

[CR28] Dudnik E, Bshara E, Grubstein A, Fridel L, Shochat T, Roisman LC, Ilouze M, Rozenblum AB, Geva S, Zer A, Rotem O, Allen AM, Peled N (2018a) Rare targetable drivers (RTDs) in non-small cell lung cancer (NSCLC): outcomes with immune check-point inhibitors (ICPi). Lung Cancer 124:117–124 30268448 10.1016/j.lungcan.2018.07.044

[CR29] Dudnik E, Peled N, Nechushtan H, Wollner M, Onn A, Agbarya A, Moskovitz M, Keren S, Popovits-Hadari N, Urban D, Mishaeli M, Zer A, Allen AM, Rabinovich NM, Rotem O, Kuznetsov T, Shochat T, Roisman LC, Bar J (2018b) BRAF mutant lung cancer: programmed death ligand 1 expression, tumor mutational burden, microsatellite instability status, and response to immune check-point inhibitors. J Thorac Oncol 13:1128–1137 29723688 10.1016/j.jtho.2018.04.024

[CR30] Ekman S (2019) HER2: defining a Neu target in non-small-cell lung cancer. Ann Oncol 30:353–355 30715217 10.1093/annonc/mdz043

[CR31] Enokida T, Moreira A, Bhardwaj N (2021) Vaccines for immunoprevention of cancer. J Clin Invest 131:e146956 33938446 10.1172/JCI146956 PMC8087198

[CR32] Fan J, Bellon M, Ju M, Zhao L, Wei M, Fu L, Nicot C (2022) Clinical significance of FBXW7 loss of function in human cancers. Mol Cancer 21:87 35346215 10.1186/s12943-022-01548-2 PMC8962602

[CR33] Fang W, Ma Y, Yin JC, Hong S, Zhou H, Wang A, Wang F, Bao H, Wu X, Yang Y, Huang Y, Zhao H, Shao YW, Zhang L (2019) Comprehensive genomic profiling identifies novel genetic predictors of response to anti-PD-(L)1 therapies in non-small cell lung cancer. Clin Cancer Res 25:5015–5026 31085721 10.1158/1078-0432.CCR-19-0585

[CR34] Fehrenbacher L, Spira A, Ballinger M, Kowanetz M, Vansteenkiste J, Mazieres J, Park K, Smith D, Artal-Cortes A, Lewanski C, Braiteh F, Waterkamp D, He P, Zou W, Chen DS, Yi J, Sandler A, Rittmeyer A (2016) Atezolizumab versus docetaxel for patients with previously treated non-small-cell lung cancer (POPLAR): a multicentre, open-label, phase 2 randomised controlled trial. Lancet 387:1837–1846 26970723 10.1016/S0140-6736(16)00587-0

[CR35] Frampton GM, Ali SM, Rosenzweig M, Chmielecki J, Lu X, Bauer TM, Akimov M, Bufill JA, Lee C, Jentz D, Hoover R, Ou SH, Salgia R, Brennan T, Chalmers ZR, Jaeger S, Huang A, Elvin JA, Erlich R, Fichtenholtz A, Gowen KA, Greenbowe J, Johnson A, Khaira D, McMahon C, Sanford EM, Roels S, White J, Greshock J, Schlegel R, Lipson D, Yelensky R, Morosini D, Ross JS, Collisson E, Peters M, Stephens PJ, Miller VA (2015) Activation of MET via diverse exon 14 splicing alterations occurs in multiple tumor types and confers clinical sensitivity to MET inhibitors. Cancer Discov 5:850–859 25971938 10.1158/2159-8290.CD-15-0285

[CR36] Freed-Pastor WA, Lambert LJ, Ely ZA, Pattada NB, Bhutkar A, Eng G, Mercer KL, Garcia AP, Lin L, Rideout WM 3rd, Hwang WL, Schenkel JM, Jaeger AM, Bronson RT, Westcott PMK, Hether TD, Divakar P, Reeves JW, Deshpande V, Delorey T, Phillips D, Yilmaz OH, Regev A, Jacks T (2021) The CD155/TIGIT axis promotes and maintains immune evasion in neoantigen-expressing pancreatic cancer. Cancer Cell 39:1342–60.e14 34358448 10.1016/j.ccell.2021.07.007 PMC8511341

[CR37] Gainor JF, Shaw AT (2013) Novel targets in non-small cell lung cancer: ROS1 and RET fusions. Oncologist 18:865–875 23814043 10.1634/theoncologist.2013-0095 PMC3720641

[CR38] Gainor JF, Shaw AT, Sequist LV, Fu X, Azzoli CG, Piotrowska Z, Huynh TG, Zhao L, Fulton L, Schultz KR, Howe E, Farago AF, Sullivan RJ, Stone JR, Digumarthy S, Moran T, Hata AN, Yagi Y, Yeap BY, Engelman JA, Mino-Kenudson M (2016) EGFR mutations and ALK rearrangements are associated with low response rates to PD-1 pathway blockade in non-small cell lung cancer: a retrospective analysis. Clin Cancer Res 22:4585–4593 27225694 10.1158/1078-0432.CCR-15-3101 PMC5026567

[CR39] Garassino MC, Cho BC, Kim JH, Mazières J, Vansteenkiste J, Lena H, Corral Jaime J, Gray JE, Powderly J, Chouaid C, Bidoli P, Wheatley-Price P, Park K, Soo RA, Huang Y, Wadsworth C, Dennis PA, Rizvi NA (2018) Durvalumab as third-line or later treatment for advanced non-small-cell lung cancer (ATLANTIC): an open-label, single-arm, phase 2 study. Lancet Oncol 19:521–536 29545095 10.1016/S1470-2045(18)30144-X PMC7771363

[CR40] Gstalder C, Liu D, Miao D, Lutterbach B, DeVine AL, Lin C, Shettigar M, Pancholi P, Buchbinder EI, Carter SL, Manos MP, Rojas-Rudilla V, Brennick R, Gjini E, Chen PH, Lako A, Rodig S, Yoon CH, Freeman GJ, Barbie DA, Hodi FS, Miles W, Van Allen EM, Haq R (2020) Inactivation of Fbxw7 impairs dsRNA sensing and confers resistance to PD-1 blockade. Cancer Discov 10:1296–1311 32371478 10.1158/2159-8290.CD-19-1416 PMC8802534

[CR41] Gu J, Zhou Y, Huang L, Ou W, Wu J, Li S, Xu J, Feng J, Liu B (2016) TP53 mutation is associated with a poor clinical outcome for non-small cell lung cancer: evidence from a meta-analysis. Mol Clin Oncol 5:705–713 28101350 10.3892/mco.2016.1057 PMC5228103

[CR42] Guisier F, Dubos-Arvis C, Viñas F, Doubre H, Ricordel C, Ropert S, Janicot H, Bernardi M, Fournel P, Lamy R, Pérol M, Dauba J, Gonzales G, Falchero L, Decroisette C, Assouline P, Chouaid C, Bylicki O (2020) Efficacy and safety of anti-PD-1 immunotherapy in patients with advanced NSCLC With BRAF, HER2, or MET mutations or RET translocation: GFPC 01–2018. J Thorac Oncol 15:628–636 31945494 10.1016/j.jtho.2019.12.129

[CR43] Hellmann MD, Ciuleanu TE, Pluzanski A, Lee JS, Otterson GA, Audigier-Valette C, Minenza E, Linardou H, Burgers S, Salman P, Borghaei H, Ramalingam SS, Brahmer J, Reck M, O’Byrne KJ, Geese WJ, Green G, Chang H, Szustakowski J, Bhagavatheeswaran P, Healey D, Fu Y, Nathan F, Paz-Ares L (2018a) Nivolumab plus ipilimumab in lung cancer with a high tumor mutational burden. N Engl J Med 378:2093–2104 29658845 10.1056/NEJMoa1801946 PMC7193684

[CR44] Hellmann MD, Nathanson T, Rizvi H, Creelan BC, Sanchez-Vega F, Ahuja A, Ni A, Novik JB, Mangarin LMB, Abu-Akeel M, Liu C, Sauter JL, Rekhtman N, Chang E, Callahan MK, Chaft JE, Voss MH, Tenet M, Li XM, Covello K, Renninger A, Vitazka P, Geese WJ, Borghaei H, Rudin CM, Antonia SJ, Swanton C, Hammerbacher J, Merghoub T, McGranahan N, Snyder A, Wolchok JD (2018b) Genomic features of response to combination immunotherapy in patients with advanced non-small-cell lung cancer. Cancer Cell 33:843–52.e4 29657128 10.1016/j.ccell.2018.03.018 PMC5953836

[CR45] Herbst RS, Soria JC, Kowanetz M, Fine GD, Hamid O, Gordon MS, Sosman JA, McDermott DF, Powderly JD, Gettinger SN, Kohrt HE, Horn L, Lawrence DP, Rost S, Leabman M, Xiao Y, Mokatrin A, Koeppen H, Hegde PS, Mellman I, Chen DS, Hodi FS (2014) Predictive correlates of response to the anti-PD-L1 antibody MPDL3280A in cancer patients. Nature 515:563–567 25428504 10.1038/nature14011 PMC4836193

[CR46] Herbst RS, Baas P, Kim DW, Felip E, Pérez-Gracia JL, Han JY, Molina J, Kim JH, Arvis CD, Ahn MJ, Majem M, Fidler MJ, deCastro G Jr, Garrido M, Lubiniecki GM, Shentu Y, Im E, Dolled-Filhart M, Garon EB (2016) Pembrolizumab versus docetaxel for previously treated, PD-L1-positive, advanced non-small-cell lung cancer (KEYNOTE-010): a randomised controlled trial. Lancet 387:1540–1550 26712084 10.1016/S0140-6736(15)01281-7

[CR47] Hu Q, Zhang B, Chen R, Fu C, J A, Fu X, Li J, Fu L, Zhang Z, Dong JT (2019) ZFHX3 is indispensable for ERβ to inhibit cell proliferation via MYC downregulation in prostate cancer cells. Oncogenesis 8:28 30979864 10.1038/s41389-019-0138-y PMC6461672

[CR48] Jamal-Hanjani M, Wilson GA, McGranahan N, Birkbak NJ, Watkins TBK, Veeriah S, Shafi S, Johnson DH, Mitter R, Rosenthal R, Salm M, Horswell S, Escudero M, Matthews N, Rowan A, Chambers T, Moore DA, Turajlic S, Xu H, Lee SM, Forster MD, Ahmad T, Hiley CT, Abbosh C, Falzon M, Borg E, Marafioti T, Lawrence D, Hayward M, Kolvekar S, Panagiotopoulos N, Janes SM, Thakrar R, Ahmed A, Blackhall F, Summers Y, Shah R, Joseph L, Quinn AM, Crosbie PA, Naidu B, Middleton G, Langman G, Trotter S, Nicolson M, Remmen H, Kerr K, Chetty M, Gomersall L, Fennell DA, Nakas A, Rathinam S, Anand G, Khan S, Russell P, Ezhil V, Ismail B, Irvin-Sellers M, Prakash V, Lester JF, Kornaszewska M, Attanoos R, Adams H, Davies H, Dentro S, Taniere P, O’Sullivan B, Lowe HL, Hartley JA, Iles N, Bell H, Ngai Y, Shaw JA, Herrero J, Szallasi Z, Schwarz RF, Stewart A, Quezada SA, Le Quesne J, Van Loo P, Dive C, Hackshaw A, Swanton C (2017) Tracking the evolution of non-small-cell lung cancer. N Engl J Med 376:2109–2121 28445112 10.1056/NEJMoa1616288

[CR49] Kadara H, Choi M, Zhang J, Parra ER, Rodriguez-Canales J, Gaffney SG, Zhao Z, Behrens C, Fujimoto J, Chow C, Yoo Y, Kalhor N, Moran C, Rimm D, Swisher S, Gibbons DL, Heymach J, Kaftan E, Townsend JP, Lynch TJ, Schlessinger J, Lee J, Lifton RP, Wistuba II, Herbst RS (2017) Whole-exome sequencing and immune profiling of early-stage lung adenocarcinoma with fully annotated clinical follow-up. Ann Oncol 28:75–82 27687306 10.1093/annonc/mdw436 PMC5982809

[CR50] Katoh M (2012) Function and cancer genomics of FAT family genes (review). Int J Oncol 41:1913–1918 23076869 10.3892/ijo.2012.1669 PMC3583642

[CR51] Kauffmann-Guerrero D, Tufman A, Kahnert K, Bollmann BA, Reu S, Syunyaeva Z, Schneider C, Manapov F, Huber RM, Golpon H (2020) Response to checkpoint inhibition in non-small cell lung cancer with molecular driver alterations. Oncol Res Treat 43:289–298 32268332 10.1159/000506842

[CR52] Kerr KM, Thunnissen E, Dafni U, Finn SP, Bubendorf L, Soltermann A, Verbeken E, Biernat W, Warth A, Marchetti A, Speel EM, Pokharel S, Quinn AM, Monkhorst K, Navarro A, Madsen LB, Radonic T, Wilson J, De Luca G, Gray SG, Cheney R, Savic S, Martorell M, Muley T, Baas P, Meldgaard P, Blackhall F, Dingemans AM, Dziadziuszko R, Vansteenkiste J, Weder W, Polydoropoulou V, Geiger T, Kammler R, Peters S, Stahel R (2019) A retrospective cohort study of PD-L1 prevalence, molecular associations and clinical outcomes in patients with NSCLC: results from the European thoracic oncology platform (ETOP) lungscape project. Lung Cancer 131:95–103 31027705 10.1016/j.lungcan.2019.03.012

[CR53] Kohno T, Ichikawa H, Totoki Y, Yasuda K, Hiramoto M, Nammo T, Sakamoto H, Tsuta K, Furuta K, Shimada Y, Iwakawa R, Ogiwara H, Oike T, Enari M, Schetter AJ, Okayama H, Haugen A, Skaug V, Chiku S, Yamanaka I, Arai Y, Watanabe S, Sekine I, Ogawa S, Harris CC, Tsuda H, Yoshida T, Yokota J, Shibata T (2012) KIF5B-RET fusions in lung adenocarcinoma. Nat Med 18:375–377 22327624 10.1038/nm.2644 PMC6430196

[CR54] Lee CK, Man J, Lord S, Cooper W, Links M, Gebski V, Herbst RS, Gralla RJ, Mok T, Yang JC (2018) Clinical and molecular characteristics associated with survival among patients treated with checkpoint inhibitors for advanced non-small cell lung carcinoma: a systematic review and meta-analysis. JAMA Oncol 4:210–216 29270615 10.1001/jamaoncol.2017.4427 PMC5838598

[CR55] Lee J, Ku BM, Shim JH, La Choi Y, Sun JM, Lee SH, Ahn JS, Park K, Ahn MJ (2020) Characteristics and outcomes of RET-rearranged Korean non-small cell lung cancer patients in real-world practice. Jpn J Clin Oncol 50:594–601 32083304 10.1093/jjco/hyaa019

[CR56] Liu CX, Li Y, Obermoeller-McCormick LM, Schwartz AL, Bu G (2001) The putative tumor suppressor LRP1B, a novel member of the low density lipoprotein (LDL) receptor family, exhibits both overlapping and distinct properties with the LDL receptor-related protein. J Biol Chem 276:28889–28896 11384978 10.1074/jbc.M102727200

[CR57] Liu XY, Cui YN, Li J, Zhang Z, Guo RH (2022) Effect of FBXW7 gene mutation on the prognosis of immunotherapy in patients with non-small cell lung cancer. Zhonghua Yi Xue Za Zhi 102:914–921 35385962 10.3760/cma.j.cn112137-20211021-02332

[CR58] Long J, Wang D, Yang X, Wang A, Lin Y, Zheng M, Zhang H, Sang X, Wang H, Hu K, Zhao H (2021) Identification of NOTCH4 mutation as a response biomarker for immune checkpoint inhibitor therapy. BMC Med 19:154 34284787 10.1186/s12916-021-02031-3 PMC8293505

[CR59] Lu S, Stein JE, Rimm DL, Wang DW, Bell JM, Johnson DB, Sosman JA, Schalper KA, Anders RA, Wang H, Hoyt C, Pardoll DM, Danilova L, Taube JM (2019) Comparison of biomarker modalities for predicting response to PD-1/PD-L1 checkpoint blockade: a systematic review and meta-analysis. JAMA Oncol 5:1195–1204 31318407 10.1001/jamaoncol.2019.1549 PMC6646995

[CR60] Mao L, Zhao ZL, Yu GT, Wu L, Deng WW, Li YC, Liu JF, Bu LL, Liu B, Kulkarni AB, Zhang WF, Zhang L, Sun ZJ (2018) γ-Secretase inhibitor reduces immunosuppressive cells and enhances tumour immunity in head and neck squamous cell carcinoma. Int J Cancer 142:999–1009 29047105 10.1002/ijc.31115

[CR61] Marabelle A, Fakih M, Lopez J, Shah M, Shapira-Frommer R, Nakagawa K, Chung HC, Kindler HL, Lopez-Martin JA, Miller WH Jr, Italiano A, Kao S, Piha-Paul SA, Delord JP, McWilliams RR, Fabrizio DA, Aurora-Garg D, Xu L, Jin F, Norwood K, Bang YJ (2020) Association of tumour mutational burden with outcomes in patients with advanced solid tumours treated with pembrolizumab: prospective biomarker analysis of the multicohort, open-label, phase 2 KEYNOTE-158 study. Lancet Oncol 21:1353–1365 32919526 10.1016/S1470-2045(20)30445-9

[CR62] Marinelli D, Mazzotta M, Scalera S, Terrenato I, Sperati F, D’Ambrosio L, Pallocca M, Corleone G, Krasniqi E, Pizzuti L, Barba M, Carpano S, Vici P, Filetti M, Giusti R, Vecchione A, Occhipinti M, Gelibter A, Botticelli A, De Nicola F, Ciuffreda L, Goeman F, Gallo E, Visca P, Pescarmona E, Fanciulli M, De Maria R, Marchetti P, Ciliberto G, Maugeri-Saccà M (2020) KEAP1-driven co-mutations in lung adenocarcinoma unresponsive to immunotherapy despite high tumor mutational burden. Ann Oncol 31:1746–1754 32866624 10.1016/j.annonc.2020.08.2105

[CR63] Mazières J, Peters S, Lepage B, Cortot AB, Barlesi F, Beau-Faller M, Besse B, Blons H, Mansuet-Lupo A, Urban T, Moro-Sibilot D, Dansin E, Chouaid C, Wislez M, Diebold J, Felip E, Rouquette I, Milia JD, Gautschi O (2013) Lung cancer that harbors an HER2 mutation: epidemiologic characteristics and therapeutic perspectives. J Clin Oncol 31:1997–2003 23610105 10.1200/JCO.2012.45.6095

[CR64] Mazieres J, Drilon A, Lusque A, Mhanna L, Cortot AB, Mezquita L, Thai AA, Mascaux C, Couraud S, Veillon R, Van den Heuvel M, Neal J, Peled N, Früh M, Ng TL, Gounant V, Popat S, Diebold J, Sabari J, Zhu VW, Rothschild SI, Bironzo P, Martinez-Marti A, Curioni-Fontecedro A, Rosell R, Lattuca-Truc M, Wiesweg M, Besse B, Solomon B, Barlesi F, Schouten RD, Wakelee H, Camidge DR, Zalcman G, Novello S, Ou SI, Milia J, Gautschi O (2019) Immune checkpoint inhibitors for patients with advanced lung cancer and oncogenic driver alterations: results from the IMMUNOTARGET registry. Ann Oncol 30:1321–1328 31125062 10.1093/annonc/mdz167 PMC7389252

[CR65] Molina JR, Yang P, Cassivi SD, Schild SE, Adjei AA (2008) Non-small cell lung cancer: epidemiology, risk factors, treatment, and survivorship. Mayo Clin Proc 83:584–594 18452692 10.4065/83.5.584 PMC2718421

[CR66] Mota STS, Vecchi L, Zóia MAP, Oliveira FM, Alves DA, Dornelas BC, Bezerra SM, Andrade VP, Maia YCP, Neves AF, Goulart LR, Araújo TG (2019) New insights into the role of polybromo-1 in prostate cancer. Int J Mol Sci 20:2852 31212728 10.3390/ijms20122852 PMC6627401

[CR67] Mullins SR, Vasilakos JP, Deschler K, Grigsby I, Gillis P, John J, Elder MJ, Swales J, Timosenko E, Cooper Z, Dovedi SJ, Leishman AJ, Luheshi N, Elvecrog J, Tilahun A, Goodwin R, Herbst R, Tomai MA, Wilkinson RW (2019) Intratumoral immunotherapy with TLR7/8 agonist MEDI9197 modulates the tumor microenvironment leading to enhanced activity when combined with other immunotherapies. J Immunother Cancer 7:244 31511088 10.1186/s40425-019-0724-8 PMC6739946

[CR68] Mumm JS, Kopan R (2000) Notch signaling: from the outside in. Dev Biol 228:151–165 11112321 10.1006/dbio.2000.9960

[CR69] Negrao MV, Skoulidis F, Montesion M, Schulze K, Bara I, Shen V, Xu H, Hu S, Sui D, Elamin YY, Le X, Goldberg ME, Murugesan K, Wu CJ, Zhang J, Barreto DS, Robichaux JP, Reuben A, Cascone T, Gay CM, Mitchell KG, Hong L, Rinsurongkawong W, Roth JA, Swisher SG, Lee J, Tsao A, Papadimitrakopoulou V, Gibbons DL, Glisson BS, Singal G, Miller VA, Alexander B, Frampton G, Albacker LA, Shames D, Zhang J, Heymach JV (2021) Oncogene-specific differences in tumor mutational burden, PD-L1 expression, and outcomes from immunotherapy in non-small cell lung cancer. J Immunother Cancer 9:e002891 34376553 10.1136/jitc-2021-002891 PMC8356172

[CR70] Parikh AR, Ali SM, Schrock AB, Albacker LA, Miller VA, Stephens PJ, Crilley P, Markman M (2018) Response to rapamycin analogs but not PD-1 inhibitors in PTEN-mutated metastatic non-small-cell lung cancer with high tumor mutational burden. Lung Cancer 9:45–47 29844707 10.2147/LCTT.S161738 PMC5963481

[CR71] Peng W, Chen JQ, Liu C, Malu S, Creasy C, Tetzlaff MT, Xu C, McKenzie JA, Zhang C, Liang X, Williams LJ, Deng W, Chen G, Mbofung R, Lazar AJ, Torres-Cabala CA, Cooper ZA, Chen PL, Tieu TN, Spranger S, Yu X, Bernatchez C, Forget MA, Haymaker C, Amaria R, McQuade JL, Glitza IC, Cascone T, Li HS, Kwong LN, Heffernan TP, Hu J, Bassett RL Jr, Bosenberg MW, Woodman SE, Overwijk WW, Lizée G, Roszik J, Gajewski TF, Wargo JA, Gershenwald JE, Radvanyi L, Davies MA, Hwu P (2016) Loss of PTEN promotes resistance to T cell-mediated immunotherapy. Cancer Discov 6:202–216 26645196 10.1158/2159-8290.CD-15-0283 PMC4744499

[CR72] Pyo KH, Lim SM, Park CW, Jo HN, Kim JH, Yun MR, Kim D, Xin CF, Lee W, Gheorghiu B, Hong MH, Kim HR, Shim HS, Jang M, Lee SS, Cho BC (2020) Comprehensive analyses of immunodynamics and immunoreactivity in response to treatment in ALK-positive non-small-cell lung cancer. J Immunother Cancer 8:e000970 32727812 10.1136/jitc-2020-000970 PMC7394300

[CR73] Ren K, Peng Q, Ding G, Yu Y, Huang T, Gong L, Yu T, Yang L (2022) Potential biomarkers and resistance mechanisms of atezolizumab in a patient with lung squamous cell carcinoma. Immunotherapy 14:15–21 34763535 10.2217/imt-2020-0325

[CR74] Rikova K, Guo A, Zeng Q, Possemato A, Yu J, Haack H, Nardone J, Lee K, Reeves C, Li Y, Hu Y, Tan Z, Stokes M, Sullivan L, Mitchell J, Wetzel R, Macneill J, Ren JM, Yuan J, Bakalarski CE, Villen J, Kornhauser JM, Smith B, Li D, Zhou X, Gygi SP, Gu TL, Polakiewicz RD, Rush J, Comb MJ (2007) Global survey of phosphotyrosine signaling identifies oncogenic kinases in lung cancer. Cell 131:1190–1203 18083107 10.1016/j.cell.2007.11.025

[CR75] Rizvi H, Sanchez-Vega F, La K, Chatila W, Jonsson P, Halpenny D, Plodkowski A, Long N, Sauter JL, Rekhtman N, Hollmann T, Schalper KA, Gainor JF, Shen R, Ni A, Arbour KC, Merghoub T, Wolchok J, Snyder A, Chaft JE, Kris MG, Rudin CM, Socci ND, Berger MF, Taylor BS, Zehir A, Solit DB, Arcila ME, Ladanyi M, Riely GJ, Schultz N, Hellmann MD (2018) Molecular determinants of response to anti-programmed cell death (PD)-1 and anti-programmed death-ligand 1 (PD-L1) blockade in patients with non-small-cell lung cancer profiled with targeted next-generation sequencing. J Clin Oncol 36:633–641 29337640 10.1200/JCO.2017.75.3384 PMC6075848

[CR76] Sabari JK, Leonardi GC, Shu CA, Umeton R, Montecalvo J, Ni A, Chen R, Dienstag J, Mrad C, Bergagnini I, Lai WV, Offin M, Arbour KC, Plodkowski AJ, Halpenny DF, Paik PK, Li BT, Riely GJ, Kris MG, Rudin CM, Sholl LM, Nishino M, Hellmann MD, Rekhtman N, Awad MM, Drilon A (2018) PD-L1 expression, tumor mutational burden, and response to immunotherapy in patients with MET exon 14 altered lung cancers. Ann Oncol 29:2085–2091 30165371 10.1093/annonc/mdy334 PMC6225900

[CR77] Schoenfeld AJ, Bandlamudi C, Lavery JA, Montecalvo J, Namakydoust A, Rizvi H, Egger J, Concepcion CP, Paul S, Arcila ME, Daneshbod Y, Chang J, Sauter JL, Beras A, Ladanyi M, Jacks T, Rudin CM, Taylor BS, Donoghue MTA, Heller G, Hellmann MD, Rekhtman N, Riely GJ (2020) The genomic landscape of SMARCA4 alterations and associations with outcomes in patients with lung cancer. Clin Cancer Res 26:5701–5708 32709715 10.1158/1078-0432.CCR-20-1825 PMC7641983

[CR78] Skoulidis F, Byers LA, Diao L, Papadimitrakopoulou VA, Tong P, Izzo J, Behrens C, Kadara H, Parra ER, Canales JR, Zhang J, Giri U, Gudikote J, Cortez MA, Yang C, Fan Y, Peyton M, Girard L, Coombes KR, Toniatti C, Heffernan TP, Choi M, Frampton GM, Miller V, Weinstein JN, Herbst RS, Wong KK, Zhang J, Sharma P, Mills GB, Hong WK, Minna JD, Allison JP, Futreal A, Wang J, Wistuba II, Heymach JV (2015) Co-occurring genomic alterations define major subsets of KRAS-mutant lung adenocarcinoma with distinct biology, immune profiles, and therapeutic vulnerabilities. Cancer Discov 5:860–877 26069186 10.1158/2159-8290.CD-14-1236 PMC4527963

[CR79] Skoulidis F, Goldberg ME, Greenawalt DM, Hellmann MD, Awad MM, Gainor JF, Schrock AB, Hartmaier RJ, Trabucco SE, Gay L, Ali SM, Elvin JA, Singal G, Ross JS, Fabrizio D, Szabo PM, Chang H, Sasson A, Srinivasan S, Kirov S, Szustakowski J, Vitazka P, Edwards R, Bufill JA, Sharma N, Ou SI, Peled N, Spigel DR, Rizvi H, Aguilar EJ, Carter BW, Erasmus J, Halpenny DF, Plodkowski AJ, Long NM, Nishino M, Denning WL, Galan-Cobo A, Hamdi H, Hirz T, Tong P, Wang J, Rodriguez-Canales J, Villalobos PA, Parra ER, Kalhor N, Sholl LM, Sauter JL, Jungbluth AA, Mino-Kenudson M, Azimi R, Elamin YY, Zhang J, Leonardi GC, Jiang F, Wong KK, Lee JJ, Papadimitrakopoulou VA, Wistuba II, Miller VA, Frampton GM, Wolchok JD, Shaw AT, Jänne PA, Stephens PJ, Rudin CM, Geese WJ, Albacker LA, Heymach JV (2018) STK11/LKB1 mutations and PD-1 inhibitor resistance in KRAS-mutant lung adenocarcinoma. Cancer Discov 8:822–835 29773717 10.1158/2159-8290.CD-18-0099 PMC6030433

[CR80] Song P, Zhang J, Zhang L (2019) Refinement of diagnosis and supporting evidence for the use of immunotherapy through sequential biopsies in a case of EML4-ALK positive lung cancer. Onco Targets Ther 12:2943–2948 31114237 10.2147/OTT.S203192 PMC6489643

[CR81] Song P, Yang D, Wang H, Cui X, Si X, Zhang X, Zhang L (2020) Relationship between the efficacy of immunotherapy and characteristics of specific tumor mutation genes in non-small cell lung cancer patients. Thorac Cancer 11:1647–1654 32342665 10.1111/1759-7714.13447 PMC7262886

[CR82] Song Z, Yang L, Zhou Z, Li P, Wang W, Cheng G, Chen R, Chang L, Zhang Y, Guan Y, Xia X, Yi X, Zhou R, Chen M (2021) Genomic profiles and tumor immune microenvironment of primary lung carcinoma and brain oligo-metastasis. Cell Death Dis 12:106 33479213 10.1038/s41419-021-03410-7 PMC7820277

[CR83] Sun H, Liu SY, Zhou JY, Xu JT, Zhang HK, Yan HH, Huan JJ, Dai PP, Xu CR, Su J, Guan YF, Yi X, Yu RS, Zhong WZ, Wu YL (2020) Specific TP53 subtype as biomarker for immune checkpoint inhibitors in lung adenocarcinoma. EBioMedicine 60:102990 32927274 10.1016/j.ebiom.2020.102990 PMC7494676

[CR84] Takeuchi K, Soda M, Togashi Y, Suzuki R, Sakata S, Hatano S, Asaka R, Hamanaka W, Ninomiya H, Uehara H, Lim Choi Y, Satoh Y, Okumura S, Nakagawa K, Mano H, Ishikawa Y (2012) RET, ROS1 and ALK fusions in lung cancer. Nat Med 18:378–381 22327623 10.1038/nm.2658

[CR85] Teng J, Zhou K, Lv D, Wu C, Feng H (2022) Case report: PTEN mutation induced by anti-PD-1 therapy in stage IV lung adenocarcinoma. Front Pharmacol 13:714408 35677433 10.3389/fphar.2022.714408 PMC9168362

[CR86] Teo MY, Seier K, Ostrovnaya I, Regazzi AM, Kania BE, Moran MM, Cipolla CK, Bluth MJ, Chaim J, Al-Ahmadie H, Snyder A, Carlo MI, Solit DB, Berger MF, Funt S, Wolchok JD, Iyer G, Bajorin DF, Callahan MK, Rosenberg JE (2018) Alterations in DNA damage response and repair genes as potential marker of clinical benefit from PD-1/PD-L1 blockade in advanced urothelial cancers. J Clin Oncol 36:1685–1694 29489427 10.1200/JCO.2017.75.7740 PMC6366295

[CR87] Thompson JC, Carpenter EL, Silva BA, Rosenstein J, Chien AL, Quinn K, Espenschied CR, Mak A, Kiedrowski LA, Lefterova M, Nagy RJ, Katz SI, Yee SS, Black TA, Singh AP, Ciunci CA, Bauml JM, Cohen RB, Langer CJ, Aggarwal C (2021) Serial monitoring of circulating tumor DNA by next-generation gene sequencing as a biomarker of response and survival in patients with advanced NSCLC receiving pembrolizumab-based therapy. JCO Precis Oncol. 10.1200/PO.20.00321 34095713 10.1200/PO.20.00321 PMC8169078

[CR88] Tian P, Zeng H, Ji L, Ding Z, Ren L, Gao W, Fan Z, Li L, Le X, Li P, Zhang M, Xia X, Zhang J, Li Y, Li W (2021) Lung adenocarcinoma with ERBB2 exon 20 insertions: comutations and immunogenomic features related to chemoimmunotherapy. Lung Cancer 160:50–58 34403912 10.1016/j.lungcan.2021.07.014

[CR89] Topalian SL, Drake CG, Pardoll DM (2015) Immune checkpoint blockade: a common denominator approach to cancer therapy. Cancer Cell 27:450–461 25858804 10.1016/j.ccell.2015.03.001 PMC4400238

[CR90] Walker CJ, Miranda MA, O’Hern MJ, McElroy JP, Coombes KR, Bundschuh R, Cohn DE, Mutch DG, Goodfellow PJ (2015) Patterns of CTCF and ZFHX3 mutation and associated outcomes in endometrial cancer. J Natl Cancer Inst. 10.1093/jnci/djv249 26330387 10.1093/jnci/djv249 PMC4643635

[CR91] Wang X, Sun Q (2017) TP53 mutations, expression and interaction networks in human cancers. Oncotarget 8:624–643 27880943 10.18632/oncotarget.13483 PMC5352183

[CR92] Wang Z, Zhao J, Wang G, Zhang F, Zhang Z, Zhang F, Zhang Y, Dong H, Zhao X, Duan J, Bai H, Tian Y, Wan R, Han M, Cao Y, Xiong L, Liu L, Wang S, Cai S, Mok TSK, Wang J (2018) Comutations in DNA damage response pathways serve as potential biomarkers for immune checkpoint blockade. Cancer Res 78:6486–6496 30171052 10.1158/0008-5472.CAN-18-1814

[CR93] Wang S, He Z, Wang X, Li H, Liu XS (2019) Antigen presentation and tumor immunogenicity in cancer immunotherapy response prediction. Elife 8:e49020 31767055 10.7554/eLife.49020 PMC6879305

[CR94] Welsh SJ, Rizos H, Scolyer RA, Long GV (2016) Resistance to combination BRAF and MEK inhibition in metastatic melanoma: Where to next? Eur J Cancer 62:76–85 27232329 10.1016/j.ejca.2016.04.005

[CR95] West HJ, McCleland M, Cappuzzo F, Reck M, Mok TS, Jotte RM, Nishio M, Kim E, Morris S, Zou W, Shames D, Das Thakur M, Shankar G, Socinski MA (2022) Clinical efficacy of atezolizumab plus bevacizumab and chemotherapy in KRAS-mutated non-small cell lung cancer with STK11, KEAP1, or TP53 comutations: subgroup results from the phase III IMpower150 trial. J Immunother Cancer. 10.1136/jitc-2021-003027 35190375 10.1136/jitc-2021-003027 PMC8862451

[CR96] Wu HX, Chen YX, Wang ZX, Zhao Q, He MM, Wang YN, Wang F, Xu RH (2019) Alteration in TET1 as potential biomarker for immune checkpoint blockade in multiple cancers. J Immunother Cancer 7:264 31623662 10.1186/s40425-019-0737-3 PMC6798429

[CR97] Yang Q, Shen R, Xu H, Shi X, Xu L, Zhang L, Fan X, Jin X (2021) Comprehensive analyses of PBRM1 in multiple cancer types and its association with clinical response to immunotherapy and immune infiltrates. Ann Transl Med 9:465 33850862 10.21037/atm-21-289 PMC8039713

[CR98] Yeh CH, Bellon M, Nicot C (2018) FBXW7: a critical tumor suppressor of human cancers. Mol Cancer 17:115 30086763 10.1186/s12943-018-0857-2 PMC6081812

[CR99] Zhang K, Hong X, Song Z, Xu Y, Li C, Wang G, Zhang Y, Zhao X, Zhao Z, Zhao J, Huang M, Huang D, Qi C, Gao C, Cai S, Gu F, Hu Y, Xu C, Wang W, Lou Z, Zhang Y, Liu L (2020) Identification of deleterious NOTCH mutation as novel predictor to efficacious immunotherapy in NSCLC. Clin Cancer Res 26:3649–3661 32241817 10.1158/1078-0432.CCR-19-3976

[CR100] Zhang J, Zhou N, Lin A, Luo P, Chen X, Deng H, Kang S, Guo L, Zhu W, Zhang J (2021a) ZFHX3 mutation as a protective biomarker for immune checkpoint blockade in non-small cell lung cancer. Cancer Immunol Immunother 70:137–151 32653938 10.1007/s00262-020-02668-8 PMC10992006

[CR101] Zhang L, Zhang T, Shang B, Li Y, Cao Z, Wang H (2021b) Prognostic effect of coexisting TP53 and ZFHX3 mutations in non-small cell lung cancer patients treated with immune checkpoint inhibitors. Scand J Immunol 94:e13087 35226388 10.1111/sji.13087

[CR102] Zhou H, Liu J, Zhang Y, Huang Y, Shen J, Yang Y, Fang W, Zhang L (2020) PBRM1 mutation and preliminary response to immune checkpoint blockade treatment in non-small cell lung cancer. NPJ Precis Oncol 4:6 32195359 10.1038/s41698-020-0112-3 PMC7075870

[CR103] Zhu M, Zhang L, Cui H, Zhao Q, Wang H, Zhai B, Jiang R, Jiang Z (2021) Co-mutation of FAT3 and LRP1B in lung adenocarcinoma defines a unique subset correlated with the efficacy of immunotherapy. Front Immunol 12:800951 35069585 10.3389/fimmu.2021.800951 PMC8770854

